# A Case of Melioidosis Presenting as Acalculous Cholecystitis

**DOI:** 10.7759/cureus.2864

**Published:** 2018-06-22

**Authors:** QinHao Jonathan Ye, Suneel Ramesh Desai, Ek Khoon Tan

**Affiliations:** 1 General Medicine, Sengkang General Hospital, Singapore, SGP; 2 Anaesthesiology, Singapore General Hospital, Singapore, SGP; 3 Hepatopancreatobiliary & Transplant Surgery, Singapore General Hospital, Singapore, SGP

**Keywords:** cholecystitis, melioidosis, burkholderia pseudomallei, acalculous cholecystitis, cholecystectomy, whitmore's disease

## Abstract

We describe a case of melioidosis presenting as acalculous cholecystitis in a middle-aged Chinese male. The patient presented with clinical features of cholecystitis and computed tomography (CT) imaging did not reveal other obvious sources of sepsis other than acalculous cholecystitis. The decision was made by the hepatobiliary team to proceed with an urgent cholecystectomy in view of patient's septic presentation. Cultures from peripheral blood and intraoperatively obtained bile fluid grew *Burkholderia pseudomallei. *The patient subsequently completed one month of meropenem, followed by another three months of eradication therapy. The patient denied soil contact in his work but he comes from a melioidosis-endemic country. He was also newly diagnosed with diabetes mellitus during his admission. We believe this to be the first reported case of melioidosis presenting as acalculous cholecystitis with a positive bile fluid culture. Urgent cholecystectomy in susceptible cases, with positive contact history or from endemic countries, might present another modality to achieve source control. Appropriate antibiotics with melioidosis coverage should be started early as well.

## Introduction

Melioidosis, also called Whitmore's disease, is caused by Burkholderia pseudomallei, which is a gram-negative aerobic rod-shaped bacterium that is soil-dwelling and endemic in the tropics [1]. The most common primary site of infection is the lung, although bloodstream and disseminated infection can occur in susceptible patients, including those with diabetes mellitus, cancer, malnourishment, and renal insufficiency. There may also be a precedent history of exposure to contaminated soil or water. Organs involved might include the liver, spleen, joints, brain, and prostate.

## Case presentation

The patient was a 55-year-old Chinese male with no nationwide records of any significant past medical history. He was a current smoker and consumed alcohol daily. He worked in the operations department of a cleaning company but there was no self-reported recent soil contact or cleaning work at construction sites.

**Figure 1:**
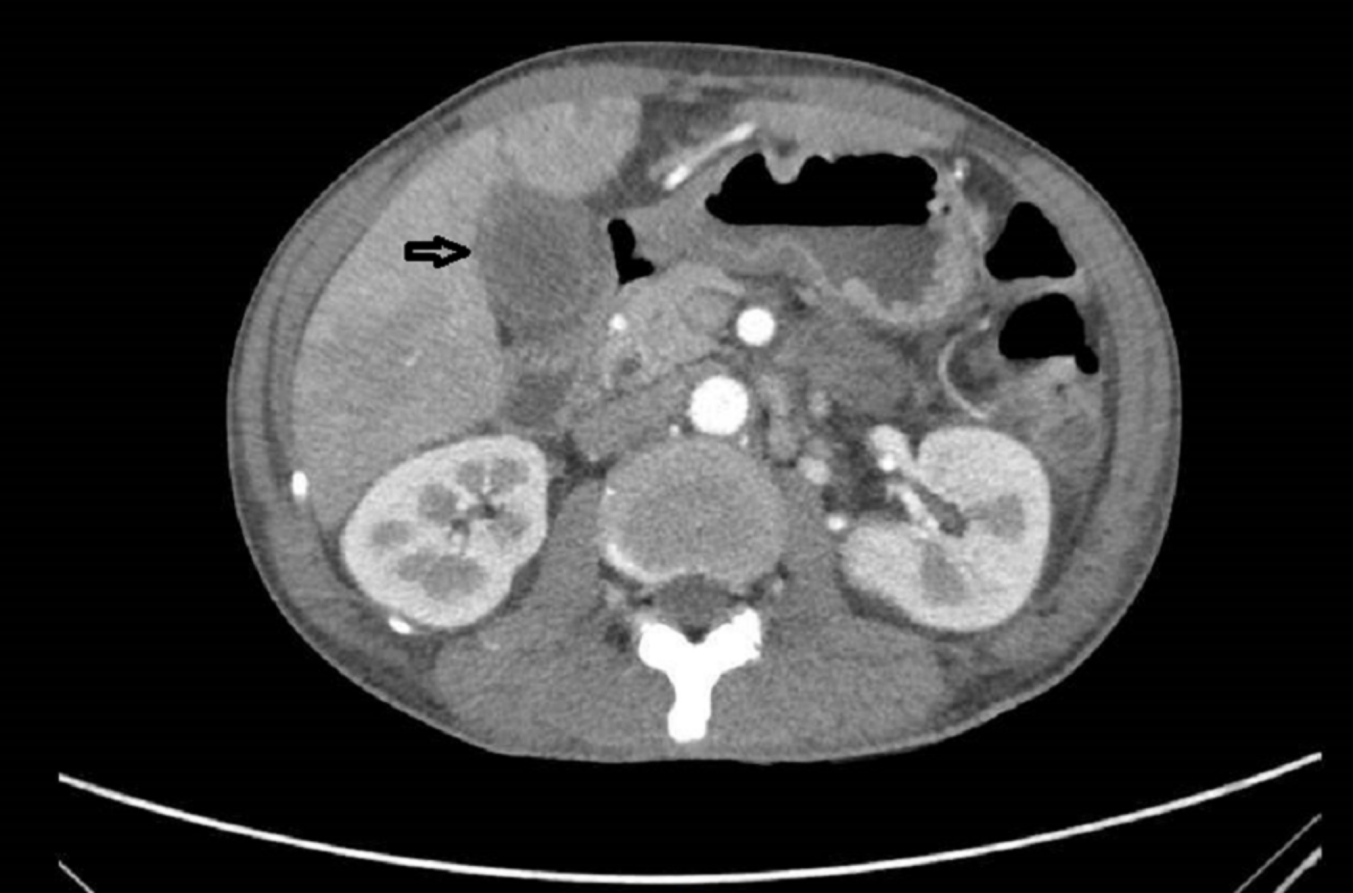
Computed tomography scan on admission The arrow indicates the location of the oedematous gallbladder.

A week prior to admission, the patient developed fever, upper abdominal pain, and yellowing of his eyes. He remained febrile on admission and was noted to be very lethargic as well. Physical examination revealed jaundice and tenderness in the right hypochondrium with a positive Murphy’s sign. Investigations revealed a raised white blood cell count of 14.4 x 109/L and a procalcitonin level of 10.7 UG/L. Serum bilirubin and alkaline phosphatase were both raised at 37 umol/L and 339 U/L, respectively. An urgent computed tomographical (CT) scan showed a diffusely thickened and oedematous gallbladder with no dilatation of the biliary tree (Figure 1). There was thrombosis seen in the right portal vein and in the splenic vein with splenic infarcts seen. There also were a few hypoenhancing foci in segment 4B/5 that could be due to ischaemia or evolving abscess. 

The reviewing surgical team made a diagnosis of severe sepsis from acute acalculous cholecystitis and decided on operative management. A laparoscopic cholecystectomy was carried out, and intraoperatively, the gallbladder was found to be distended, inflamed, and containing turbid bile. No gallstones were found in the extirpated specimen. Postoperatively, the patient developed septic shock and multiorgan failure requiring mechanical ventilation and inotropic support in the intensive care unit (ICU). Recognizing the unusual presentation of cholecystitis, antibiotic therapy was escalated postoperatively from ceftriaxone and metronidazole to meropenem. Two days later, culture results from the peripheral blood and intraoperative bile fluid grew *Burkholderia pseudomallei*.

It was established later that he had undiagnosed diabetes mellitus with a glycated haemoglobin of 10.5%. He was discharged from the ICU after a prolonged stay of almost a month and suffered additional complications of dry gangrene of his hands and feet from the severe sepsis and high inotropic requirements. He was continued on intravenous meropenem for a month before converting to enteral trimethoprim and sulfamethoxazole for another three months.

## Discussion

We believe that this is the first reported case of melioidosis manifesting as acalculous cholecystitis, with no other focus of infection. A PubMed search using the terms “melioidosis” and “cholecystitis” did not reveal any reports of patients with the gallbladder as the primary site of infection. There is, however, one case report of melioidosis presenting with acute cholangitis [2]. However, the described case did not have a positive culture from biliary fluid and interval cholecystectomy that was done two months after presentation did not have any visible bacteria seen in the resected specimen. We think that the reason behind the rarity of the gallbladder as the primary site of infection might be the relative lack of blood supply when compared with that of the common sites, such as the lungs and liver.

The mortality rate of melioidosis is especially high if appropriate antibiotics are not given and source control (if required) is not achieved early [3]. The mainstay of the treatment of melioidosis is an antibiotic therapy which is divided into two phases  - the intensive phase, followed by the eradication phase [4]. However, source control might be equally important if the source is easily resectable or drainable. Various source control methods have been described, including splenectomy [5] and drainage of abscesses, which are usually challenging as the abscesses are usually small and multiple. Cholecystectomy, when the primary source is the gallbladder, might present another modality for source control.

We present this case to highlight that melioidosis can rarely present as acalculous cholecystitis. In the severe form, it can be complicated by septic shock and multiorgan failure. Urgent cholecystectomy, rather than delayed cholecystectomy, in such situations, would be indicated for source control. In our patient, the primary site of infection was ascribed to be the gallbladder due to the clinical and intraoperative findings consistent with acute cholecystitis and the corroborative positive cultures of bile samples. Sputum cultures were negative and imaging did not reveal any abscess formation within other solid organs, such as the liver, spleen, prostate, or lung, that could have been the primary site of infection. 

Melioidosis is endemic in Singapore and other Southeast Asian countries. The above-mentioned patient could have been affected during the course of his work or during his mandatory national service. All Singaporean adult men need to serve in the military for 2.5 years and for weeks each year for another 10 years as part of the national service, and soil contact may be frequent. Reactivation of latent melioidosis from the patient's military service may be a possibility and has been described in case reports [6-7]. The patient also has the risk factors of non-diagnosed diabetes mellitus and daily alcohol consumption. 

## Conclusions

We hope that this will bring attention to the possibility of melioidosis as the infective etiology in patients presenting with severe acute acalculous cholecystitis, particularly in susceptible patients and in endemic countries (like Singapore) where this case occurred. Prompt surgery and delivery of appropriate antibiotics would be life-saving. 
